# Subclavian central venous catheter-related thrombosis in trauma patients: incidence, risk factors and influence of polyurethane type

**DOI:** 10.1186/cc12748

**Published:** 2013-05-29

**Authors:** Ariane Gentile, Laurent Petit, Françoise Masson, Vincent Cottenceau, Josseline Bertrand-Barat, Geneviève Freyburger, Catherine Pinaquy, Alain Léger, Jean-François Cochard, François Sztark

**Affiliations:** 1 Service d'Anesthésie et de Réanimation 1, Hôpital Pellegrin, Centre Hospitalier Universitaire de Bordeaux, 33076 Bordeaux Cedex, France; 2 Fédération d'anesthésie-réanimation, urgences, chirurgie ambulatoire, Hôpital d'Instruction des Armées Robert Picqué, 351 route de Toulouse, 33140 Villenave-d'Ornon, France; 3 Département de Matériovigilance, Hôpital Pellegrin, Centre Hospitalier Universitaire de Bordeaux, 33076 Bordeaux Cedex, France; 4 Laboratoire d'Hématologie Hôpital Pellegrin, Centre Hospitalier Universitaire de Bordeaux, 33076 Bordeaux Cedex, France; 5 Université de Bordeaux, INSERM, Adaptation cardiovasculaire à l'ischémie, U1034, F-33600 Pessac, France

**Keywords:** central venous catheter, upper extremity deep vein thrombosis, risk factors, multiple trauma, intracranial hypertension

## Abstract

**Introduction:**

The incidence of deep venous thrombosis (DVT) related to a central venous catheter varies considerably in ICUs depending on the population included. The aim of this study was to determine subclavian central venous catheter (SCVC)-related DVT risk factors in severely traumatized patients with regard to two kinds of polyurethane catheters.

**Methods:**

Critically ill trauma patients needing a SCVC for their usual care were prospectively included in an observational study. Depending on the month of inclusion, patients received one of the two available products in the emergency unit: either an aromatic polyurethane SCVC or an aliphatic polyurethane SCVC. Patients were screened weekly by ultrasound for SCVC-related DVT. Potential risk factors were collected, including history-related, trauma-related and SCVC-related characteristics.

**Results:**

A total of 186 patients were included with a median Injury Severity Sore of 30 and a high rate of severe brain injuries (21% of high intracranial pressure). Incidence of SCVC-related DVT was 37% (95% confidence interval: 26 to 40) in patients or 20/1,000 catheter-days. SCVC-related DVT occurred within 8 days in 65% of cases. There was no significant difference in DVT rates between the aromatic polyurethane and aliphatic polyurethane SCVC groups (38% vs. 36%). SCVC-related DVT independent risk factors were age >30 years, intracranial hypertension, massive transfusion (>10 packed red blood cell units), SCVC tip position in the internal jugular or in the innominate vein, and ipsilateral jugular catheter.

**Conclusion:**

SCVC-related DVT concerned one-third of these severely traumatized patients and was mostly clinically silent. Incidence did not depend on the type of polyurethane but was related to age >30 years, intracranial hypertension or misplacement of the SCVC. Further studies are needed to assess the cost-effectiveness of routine screening in these patients in whom thromboprophylaxis may be hazardous.

## Introduction

The incidence of central venous catheter (CVC)-related deep venous thrombosis (DVT) varies considerably in the ICU, depending on the population included and the detection methods [1,2]. The rate of subclavian central venous catheter (SCVC)-related DVT found by routine Doppler ultrasound in the literature ranges from 4 to 67% with a mean incidence of 30%, of whom only 2% were symptomatic [3]. Several thrombotic risk factors have been identified and are related either to the patient's condition or to the catheter [4].

With regard to insertion sites, a femoral insertion is known to increase the risk of infection or thrombosis. The incidence of CVC-related DVT in the upper extremities is estimated to be 2 to 6% when symptomatic and 11 to 19% when asymptomatic [5]. In their conclusion, the experts of the Cochrane review group stated that it is probably reasonable to prefer subclavian access to femoral access [6]. The CVC equipment may also be a risk factor. The incidence of thrombosis is higher with a CVC made of polyethylene than one made of polyurethane [7]. Most CVC are now composed of polyurethane. In our hospital, two brands are available and both are made of polyurethane. One CVC is made of aromatic polyurethane (Ar) and is the most commonly used. Our Medical Devices Vigilance has collected reports about thrombosis associated with its use. Another brand is made of aliphatic polyurethane (Al). This Al CVC is marketed as being more thermosensitive than Ar, so its thrombogenic potential is theoretically lower.

Trauma patients have specific risk factors and the use of anticoagulant is often problematic in the initial phase because of the risk of bleeding. Studies in trauma patients have found widely varying rates of overall DVT from 0.36 to 58% depending on the patients studied and the means used to diagnose DVT [8-10]. However, most studies in the specific setting of trauma patients are not limited to the ICU.

The aim of this study was to identify SCVC-related DVT risk factors in ICU trauma patients and to determine whether the type of polyurethane used could be an independent risk factor. The study was performed regardless of the patient's clinical signs with systematic venous ultrasound screening.

## Materials and methods

### Patients

This prospective, single-center study took place in a 25-bed surgical trauma ICU at Bordeaux University Hospital. This level 1 trauma center, serving the southwest of France (2.8 million inhabitants), takes care especially of severe brain or spinal cord injuries. The institutional review board at the University Hospital of Bordeaux approved the study and waived the need for informed consent because the study was observational and did not interfere in the treatment of patients (IRB du CHU de Bordeaux; Chairman: M. Leroy; Reference: JPL/JB/GD/1317/2008/RC).

We included adult patients (≥18 years), with trauma (Injury Severity Sore (ISS) >8), needing at least one CVC for an estimated length >5 days. Subclavian insertion was the only access route considered in this study. We excluded patients with known DVT risk factors, such as thromboembolic history, thrombophilia, malignancy, patients with anticoagulant treatment at the time of their injury, and patients needing immediate therapeutic anticoagulation for their injury.

### Catheters

Two kinds of catheter are available in our hospital: an Ar SCVC (Blue FlexTip^® ^catheter; Arrow International, Reading, PA, 19605 USA) or an Al SCVC (Seldiflex^®^; Prodimed-Plastimed, F95130 LePlessis Bouchard, France), To test the kind of CVC as an independent risk factor, the type of available CVC (Ar or Al) was established on a monthly randomized basis.

### Deep venous thrombosis screening

DVT screening of the upper extremities was performed by venous duplex ultrasound scanning as a preliminary study to set up a new systematic protocol of DVT screening in our ICU. Examinations took place first between 5 and 7 days after trauma occurrence and then weekly, with the last one at the time of SCVC ablation or a few days after. The ultrasound examinations were performed by two US-licensed anesthesiologists (LP, VC) using an Acuson CV70, Siemens Medical Solution, Mountain View, CA 94043 USA with a high-frequency transducer dedicated to vascular imaging (5 to 10 MHz). A DVT was defined as partial if the vein was still partly compressible, or as complete when no venous flow could be detected. Thrombosis was considered SCVC related when a partial or complete thrombus was found in the subclavian or axillary or humeral veins with the SCVC in this territory. The examination concerned the superior vena caval territory ipsilateral and contralateral to the SCVC and the lower limbs (femoral vein up to popliteal vein).

### Data collection

The following data were collected: age, sex, date of admission, comorbidities, injury mechanism, and Simplified Acute Physiologic Score II. Injuries were recorded and their severity coded according to the Abbreviated Injury Score (AIS) maximum for each body region and to the ISS. Other data were collected concerning difficulties at the time of insertion, parenteral nutrition, medications infused through the catheter, transfusion, microbiologically documented concomitant infections (bacteremia or pneumonia), length of catheter maintenance, reason for removal, and results of catheter culture.

Coagulation screening tests performed at the time of each examination included standard tests (prothrombin time, activated partial thromboplastin time, platelet count) and specific assays for D-dimer, fibrinogen, factor II, factor V, and factor X.

A clinical examination was performed by a senior medical doctor before the first ultrasound examination in order to determine whether a DVT was likely or very likely based upon signs such as warmth, edema and collateral circulation.

### Statistical analysis

The SCVC type was considered the main potential risk factor. A preliminary study showed that the Ar-SCVC-related DVT incidence was 27%. A sample size of 112 patients in each group would make it possible to detect a 15% difference in the percentage of DVT incidence (considered clinically significant) with a power of 80%.

Quantitative data are presented as mean ± standard deviation or as median with 25 to 75th quartiles (interquartile range (IQR)) according to their distribution. Comparisons between patients were performed using the chi-square test or Fisher's exact test as appropriate, and the Student *t *test or the Mann-Whitney test for quantitative variables according to their distribution. The value of the clinical examination to determine a SCVC-related DVT was estimated by calculating positive and negative predictive values. Rates were calculated in patients and also reported as DVTs/1,000 catheter-days. Univariate and multivariate logistic regression analysis were performed to identify independent risk factors for development of SCVC-related DVT. All variables associated with a SCVC-related DVT risk with *P <*0.20 in the univariate analysis were investigated using multivariate analysis, with SCVC-related DVT as the dependent factor and other factors as the independent factors. Statistical significance was set at *P *<0.05 for all analysis.

## Results

Four hundred and eighty-six patients were screened during 21 months. Two hundred and ninety-five patients were not included for various reasons: cancer history, associated anticoagulation, CVC set up before transfer to our hospital, CVC in a femoral vein, estimated required duration of CVC <5 days, or logistic reasons (impossible to follow more than 10 patients at the same time on a weekly basis). Five patients were secondarily excluded (early death or early CVC removal). Finally, 186 patients were included in the analysis (84 in the Ar group and 102 in the Al group). Most were male (80%). Known DVT risk factors related to the patients' medical history were uncommon (Table 1). The median ISS was 30, meaning a majority of polytraumatized patients. The rate of severe brain-injured patients was high. One-half of the included patients had an initial Glasgow Coma Score <8, 37% of patients had a maximum head AIS equal to 5, and 21% developed severe intracranial hypertension (ICHT), one-half of them needing barbiturates to control their ICHT. The median length of stay was 20 days (IQR: 13 to 31) and the median length of catheterization was 13 days (IQR: 8 to 19). The CVC were mostly three lumina (16G/18G/18G), with a diameter of 7 Fr and a length of either 15 or 20 cm, depending on the placement side, respectively right or left.

**Table 1:** Demographic and medical characteristics of included patients and between-group comparison according to catheter polyurethane type

Characteristic	All patients (*n *= 186)	Ar SCVC (*n *= 84)	Al SCVC (*n *= 102)
Demographic characteristics			
Age (years)	38 ± 16	39 ± 16	37 ± 16
Weight (kg)	79 ± 16	78 ± 15	79 ± 16
Sex (male)	150 (81%)	67 (80%)	83 (81%)
History			
Hypertension	13 (6%)	7 (9%)	6 (6%)
Diabetes	6 (3%)	2 (2%)	4 (4%)
Smokers	71 (43%)	34 (44%)	37 (42%)
Scores			
SAPS II	34 (24 to 43)	32 (24 to 43)	33 (24 to 41)
ISS	29 (25 to 38)	29 (25 to 38)	30 (25 to 38)
Head AIS ≥3	131 (70%)	61 (73%)	70 (68%)
Chest AIS ≥3	91 (49%)	37 (44%)	54 (53%)
Abdominal AIS ≥3	31 (17%)	12 (14%)	19 (19)%
Pelvic AIS ≥3	47 (25%)	20 (24%)	27 (26%)
Spine AIS ≥3	38 (20%)	17 (20%)	21 (21%)
Injuries and treatments			
Upper extremity injury	53 (28%)	24 (29%)	29 (28%)
GCS <8	84 (40%)	34 (49%)	40 (48%)
CHT	39 (21%)	18 (21%)	21 (21%)
Barbiturates	23 (12%)	9 11%)	14 (14%)
Transfusion	65 (35%)	23 (27%)	42 (41%)

Results presented as mean ± standard deviation, as median (25th to 75th quartiles) or as number of patients (%). AIS, Abbreviated Injury Scale; Al, aliphatic polyurethane; Ar, aromatic polyurethane; GSC, Glasgow Coma Score; ICHT, intracranial hypertension; ISS, Injury Severity Score; SAPS II, Simplified Acute Physiologic Score II on ICU admission; SCVC, subclavian central venous catheter. No statistically significant difference between the two groups.

### Subclavian central venous catheter-related DVT incidence

Sixty-two patients developed a DVT diagnosed while the SCVC was in place (incidence 33%) and seven more were found 1 to 5 days after ablation of the SCVC (overall incidence 37%; 95% confidence interval: 26 to 40) (Table 2). The incidence was 64% in patients with ICHT and 30% in other trauma patients. In medullar-injured patients (AIS 4 or 5 in the spine region), the incidence was 27%. One-third of these DVTs were occlusive. One-half of the CVC-related DVTs involved only one vein, mostly the jugular vein, which was involved in 61% of DVT, and one-half involved at least two veins. Most were diagnosed early at the first ultrasound examination (65%): 44 were found at the end of the first week (24% of screened SCVCs), 16 at the end of the second week (16% of the 105 free SCVCs screened) and nine (23% on the 39 free SCVCs screened) at the end of the third week. These thromboses were mostly subclinical. The positive predictive value of the clinical examination was 29% and the negative predictive value was 74%. When considering only complete thrombosis (with an occluded vein), only two out of 10 were considered 'very likely thrombosed'.

**Table 2:** Demographic and medical characteristics of included patients and between-group comparison according to catheter type

Characteristic	Total (*n *= 186)	Ar SCVC (*n *= 84)	Al SCVC (*n *= 102)
DVT on SCVC in place	62 (33%)	29 (35%)	33 (32%)
DVT after SCVC removal	7 (4%)	3 (4%)	4 (4%)
Total SCVC-related DVT	69 (37%)	32 (38%)	37 (36%)
Diagnosed at first examination	44 (64%)	18 (56%)	26 (70%)
Extent			
1 vein	37 (20%)	15 (18%)	22 (22%)
2 veins	17 (9%)	8 (9%)	9 (9%)
>2 veins	15 (8%)	9 (11%)	6 (6%)
Occlusive character	25 (13%)	15 (18%)	11 (11%)

Results presented as number of patients (%). Al, aliphatic polyurethane; Ar, aromatic polyurethane; DVT, deep vein thrombosis; SCVC, subclavian central venous catheter. No statistically significant difference between the two groups.

### Associated thrombosis

Ten upper-limb DVTs were found in patients without an ipsilateral SCVC: in five cases the two sides were thrombotic, two patients had a jugular catheter on the side of the thrombosis and three occurred only on the opposite side that was free of any catheter.

There were 22 cases of above-knee DVT in the 186 patients (12%) and the femoral vein was involved in 20 out of 22. They also occurred early: 45% were found at the first ultrasound examination. Thirteen occurred in patients with SCVC-related DVT (19% in patients with a SCVC-related DVT and 8% among those who were free, *P *<0.05). They were found at the same examination in six cases, before SCVC-related DVT in two cases and after SCVC-related DVT in five cases. The overall incidence of thrombosis in this sample of very severe trauma patients was thus 44%.

### Type of catheter as risk factor

The Ar group and the Al group were comparable concerning demographic and trauma characteristics (Table 1). The overall rate of SCVC-related DVT was 38% in the Ar group and 36% in the Al group (*P *= 0.93) (Table 2).

### Other patient-related risk factors

Mean age was not statistically different between the two groups (Table 3). However, the incidence of DVT was lower in patients ≤30 years old (25.7%) than in those between 31 and 50 years old (47%) or >50 years old (40%) (*P *<0.05). SCVC-related DVT risk factors in relation to trauma characteristics are shown in Table 4. ICHT and treatment with barbiturates were significantly associated with a higher risk of SCVC-related DVT. Other head injuries, chest or abdominal or spinal cord or limb injuries did not appear associated with an increased risk of SCVC-related DVT. The rate did not depend on the ISS (Table 4) or on the number of severely injured regions (AIS ≥3) of the body (36% with one region, 35% with two regions, 42% with three or four regions). Neither patients needing a cervical collar nor those suffering from a clavicle or humerus fracture developed more SCVC-related DVTs, although a worsened venous return might be expected in such settings. Eight patients needed more than 10 packed red blood cells and six out of eight developed a thrombosis on their CVC. There was a tendency for CVC thrombosis risk in patients requiring treatment with vasopressor amines. A jugular venous catheter was inserted on the same side as the SCVC in 19 patients, including 15 with SCVC-related DVT (79%).

**Table 3:** Characteristics of patients with and without subclavian venous central catheter-related deep venous thrombosis

Characteristic	SCVC-related DVT (*n *= 69)	Not SCVC-related DVT (*n *= 117)
Demographic characteristics		
Sex (male)	54 (78%)	96 (82%)
Age (years)	40 ± 14	37 ± 17
Weight (kg)	78 ± 14	80 ± 17
Height (cm)	172 ± 14	174 ± 11
Clinical history		
Obesity^a^	12 (17%)	24 (20%)
Hypertension	6 (9%)	7 (6%)
Diabetes	1 (1%)	5 (4%)
Smokers	26 (40%)	45 (44%)
Sepsis during catheterization length		
Bacteraemia	1 (1%)	8 (7%)
Pneumonia	40 (58%)	72 (62%)

Results presented as mean ± standard deviation or as number of patients (%). Percentages are calculated from the number of patients for which data are available. DVT, deep vein thrombosis; SCVC, subclavian central venous catheter. No statistically significant difference between the two groups. ^a^Obesity defined by body mass index >30.

**Table 4:** Trauma characteristics in patients with and without subclavian venous central catheter-related deep venous thrombosis

Characteristic	SCVC-related DVT (*n *=69)	Not SCVC-related DVT (*n *=117)
Scores		
SAPS II	36 (26-46)	32 (23-39)
ISS	31 (25-38)	29 (25-36)
AIS ≥ 3		
Head	52 (75%)	79 (67%)
Chest	38 (55%)	53 (45%)
Abdominal	10 (14%)	21 (18%)
Pelvic/lower extremities	14 (20%)	33 (28%)
Spine	14 (20%)	24 (20%)
Injuries and clinical complications		
Ipsilateral humerus fracture	2 (3%)	8 (7%)
Ipsilateral clavicle fracture	1 (1%)	5 (4%)
Surgery on first day	22 (32%)	50 (43%)
GCS <8	37 (54%)	46 (39%)
Intracranial hypertension	29 (42%)	15 (13%)**
Acute respiratory distress syndrome	9 (13%)	14 (12%)
Treatments		
Barbiturates	14 (20%)	9 (8%)*
Jugular catheter on same side as SCVC	15 (22%)	4 (3%)***
Transfusion	23 (33%)	42 (36%)
Packed red blood cells *n *>10	6 (9%)	2 (2%)*
Vasopressor amines	56 (81%)	80 (68%)
Cervical collar	13 (19%)	27 (23%)
Surgical intervention in first 24 hours	23 (33%)	56 (48%)

Results presented as median (25th to 75th quartiles) or as number of patients (%). AIS, Abbreviated Injury Scale; DVT, deep vein thrombosis; GCS, Glasgow coma score; ISS, Injury Severity Score; SAPS, Simplified Acute Physiologic Score; SCVC, subclavian central venous catheter. *P <0.05; **P <0.01; ***P <0.001.

At the first examination, no difference could be found in coagulation standard tests between patients with or without a CVC-related DVT. D-dimers were very high in both groups (4,696 ± 2,730 ng/ml in DVT-negative patients vs. 5,034 ± 2,727 ng/ml in DVT-positive patients). Finally, a more prolonged inflammatory status seemed to be associated with a higher risk of SCVC-related DVT. At the first examination, the mean C-reactive protein was 160 ± 89 mg/l in patients whose examination showed a thrombosis and 151 ± 79 mg/l in the other patients (*P *= NS). In patients who were free of DVT at the first examination, the mean C-reactive protein at the end of the second week was 153 ± 87 mg/l in patients who had a thrombosis at this second examination versus 82 ± 61 mg/l in patients still unaffected (*P *<0.005). We found no relationship between the C-reactive protein level measured weekly during the first 3 weeks and the occurrence of pneumonia or septicemia during the duration of catheterization. Nor did we find any relation between the highest C-reactive protein level during the study in each patient and the occurrence of any infectious episode during the same period.

Introduction of thromboprophylaxis (subcutaneous low molecular weight heparin (LMWH)) or curative heparin (mainly LMWH) was decided on a case-by-case basis. Thromboprophylaxis was prescribed in 117 patients during the duration of the study with a median delay of 13 days after the trauma. This delay was different between brain-injured and other patients: 15 ± 6 days when maximum head AIS >2 (thus including significant intracerebral injuries) versus 11 ± 6 days in other patients. Five patients developed SCVC-related DVT while anticoagulant therapy or prophylaxis was ongoing: for 5 days in one patient and for >10 days in four others (including one who was treated by heparin infusion for a femoral thrombosis). The rate of SCVC-related DVT was 41% in patients without any prophylaxis during their follow-up versus 7.5% in patients with heparin prophylaxis (*P *<0.001). SCVC-related DVT occurring while the patients were receiving LMWH occurred later (median 16 days; IQR: 9 to 21) than in SCVC-related DVT without prophylaxis (median 7 days; IQR: 6 to 11) days). In the event of SCVC-related DVT, anticoagulant treatment was given immediately in 17 cases and later in 25 others.

### Risk factors related to subclavian central venous catheter

Forty-three patients received two SCVCs and three patients received three SCVCs. Two patients developed two SCVC-related DVTs during their stay. Thus 71 SCVC-related DVTs occurred among the 235 SCVCs for a total of 3,493 days of catheterization (20 SCVC-related DVTs/1,000 days).

The rate of DVT did not depend on the number of lumens (Table 5). A SCVC tip misplaced in the internal jugular or innominate vein was significantly associated with an increased risk of SCVC-related DVT. The SCVC tip was misplaced and not removed in 20 cases (9%): SCVC thrombosis occurred in 65% of these cases within 6 days instead of 27% in patients were SCVC tip was correctly placed (*P *<0.0001). Administration of parenteral nutrition was associated with a higher incidence of SCVC thrombosis while the rate of SCVC-related DVT was lower in patients who received propofol via the SCVC. The occurrence of SCVC-related DVT was not different in patients with a septic episode during catheterization: 28% of DVTs if pneumonia during catheterization versus 33% if not; 11% if bacteremia versus 31% if not.

**Table 5:** Subclavian venous central catheter characteristics in relation to deep venous thrombosis

Characteristic	Total (*n *= 235)	SCVC-related DVT (*n *=71)	Not SCVC-related DVT (*n *=164)
Placement procedure			
Placement in emergency room	171 (73%)	55 (77%)	116 (71%)
Left-sided	158 (67%)	52 (73%)	105 (64%)
SCVC characteristics			
Number of lumina			
2	11 (5%)	3 (4%)	8 (5%)
3	199 (85%)	60 (86%)	137 (84%)
>3	25 (11%)	7 (10%)	18 (11%)
Misplaced SCVC tip	20 (9%)	13 (19%)	7 (4%)**
Colonization^a^	6 (2.5%)	2 (2.9%)	4 (2.5%)
Treatment delivered via SCVC			
Propofol	142 (61%)	35 (50%)	107 (65%)*
Parenteral nutrition	90 (38%)	34 (49%)	56 (34%)*

Results presented as number of SCVCs (%). Percentages are calculated from the number of SCVCs for which data are available. DVT, deep vein thrombosis; SCVC, subclavian central venous catheter. ^a^SCVC colonization (≥10^3 ^colony-forming units/ml) - data available on 225 SCVCs. *P <0.05; **P <0.01.

Upon removal, the catheter tips were systematically collected for bacteriological analysis. This analysis was available in 225 cases: catheter colonization (≥10^3 ^colony-forming units/ml) was found in 2.9% of thrombotic SCVCs versus 2.5% without (*P *= NS). Colonization did not depend on the length of catheterization (15 ± 11days in colonized SCVCs vs. 14 ± 9 days in others). Among the 71 SCVC-related DVTs, 18% were found after SCVC removal; 38% SCVCs still in place were removed within 0 to 3 days after discovery of SCVC-related DVT, while 48% were left in place longer.

### Multivariate analysis

In the multivariate analysis, age was analyzed in two groups: ≤30 years old and >30 years old. The independent factors associated with SCVC-related DVT were age >30 years, ICHT, transfusion >10 packed red blood cell units, misplaced SCVC tip position and LMWH before DVT (Table 6).

**Table 6:** Risk factor predictors of subclavian venous central catheter-related deep venous thrombosis

Predictor	Univariate analysis	Multivariate analysis
Age (per year of age)	1.01 (0.99 to 1.03)^†^	
Age >30 years	2.3 (1.2 to 4.4)**	3.6 (1.6 to 8.0)**
Injuries and treatments		
Intracranial hypertension	4.9 (2.4 to 10.2)***	6.1 (2.6 to 14.4)***
Packed red blood cells *n *>10	2.7 (0.7 to 9.9)^†^	8.3 (1.7 to 40.0)*
Vasopressor amines	2.0 (0.9 to 4.1)^†^	
Parenteral nutrition	1.7 (0.8 to 3.2)^†^	
Initial surgical intervention	0.5 (0.3 to 1.0)^†^	
LMWH	0.11 (0.04 to 0.3)***	0.1 (0.02 to 0.2)***
SCVC characteristics		
Misplaced tip position	4.7 (1.7 to 12.9)***	10.2 (2.8 to 37.0)*
Ipsilateral jugular SCVC	6.0 (2.6 to 16.2)***	

Results presented as odds ratio (95% confidence interval). In patients with subclavian central venous catheter (SCVC)-related deep venous thrombosis (DVT), only low molecular weight heparin (LMWH) prescribed before DVT was considered. ^†^*P *<0.20; *P <0.05; **P <0.01; ***P <0.001.

### Short-term outcome of subclavian central venous catheter-related DVT

Forty-nine SCVC-related DVTs were revised after the date of thrombosis. SCVC-related DVTs disappeared in 23 cases (47%). Healing was observed in 50% of cases where the SCVC was left in place and 44% where it was removed (*P *= NS). Healing had occurred at the last examination in 12 out of 27 (44%) patients with LWMH and in 11 out of 22 (50%) patients without this treatment (*P *= NS). Two patients presented a pulmonary embolism (PE) confirmed by radiological imagery. One was found to have occlusive thrombi both around the SCVC and in the leg. This patient was treated with heparin and the thrombi disappeared. He had a PE 20 days later. In another patient no thrombus was found at the time of the last ultrasound examination when the CVC was removed. A PE was then diagnosed 4 days later and another ultrasound examination showed an occlusive thrombus at the site of the former SCVC. A PE was suspected in three more patients who presented a brief hypoxic episode at the time of CVC removal. Lastly, a patient suddenly died 1 month after having developed a CVC thrombosis, despite receiving curative anticoagulant treatment. This could have been due to a PE, although no radiological examination was available to confirm this hypothesis. One patient was discharged with occlusive SCVC-related DVT under anticoagulant therapy. She was hospitalized again 1 year later and the DVT was still occlusive.

## Discussion

The incidence of subclavian SCVC-related DVT concerned one-third of these patients. The hypothesis that Ar might lead to more thrombosis than Al is not confirmed. Only 186 patients were included in the study instead of the 224 originally planned. The clinical impress during the study was equivalence between the two products. An independent statistical team was consulted and found that the difference in SCVC-related DVT incidence between the Ar group and the Al group was as low as 2%. The number needed to have a good probability to demonstrate that this difference was significant should have been too high. Because such a small difference was not expected to have any impact on clinical practice, we decided to stop the study.

Other SCVC characteristics, such as multiple lumina and left-side SCVC placement, also did not appear associated with a higher risk of SCVC-related DVT in trauma patients, although they have been reported in other studies [11-13]. Parenteral nutrition was associated with a higher rate of SCVC-related DVT. Propofol seemed to have an opposite effect. Parenteral nutrition may have some impact on coagulation parameters [14]. We did not find any report of a protective effect of propofol. In our study these relations might be due to a statistical bias because patients who received parenteral nutrition had a higher frequency of ICHT while those sedated with propofol were the least severely injured. These two factors did not remain significant in the multivariate analysis. A CVC in the internal jugular vein ipsilateral to the SCVC was an independent risk factor for thrombosis. These jugular catheters were mostly set up to monitor cerebral blood saturation, which required retrograde cannulation into the jugular bulb. The analysis confirmed the high risk of not centrally positioning the tip of the subclavian CVC [8,15]. The CVC placement conditions in our emergency room may also explain the high rate of SCVC-related DVT [16].

Our results also confirm the recent report that massive transfusion (defined as >10 packed red blood cells. seems to favor SCVC-related DVT, although no threshold has been identified to date [17]. In this study there was no evidence of a relationship between infection during catheterization time and SCVC-related DVT. The infection rate was high but consisted mostly of pneumonia. Bacteremia was rare and occurred only once in association with SCVC-related DVT and that very low incidence may have a bearing on our results. Raad and colleagues found a relationship between mural thrombosis found at postmortem examination and catheter-related septicemia, but catheterization was of long duration before the examination (mean 63 days) and all patients had cancer [18]. Timsit and colleagues found that the relative risk of catheter-related septicemia was threefold higher when a thrombosis was present in critically ill patients [19]. The duration of catheterization was comparable with our study while their rate of colonization was >20%. However, 35% of their patients were admitted with an ongoing infectious disease [19]. Our DVT occurred very early and seemed to be associated more with inflammation due to trauma than to intercurrent infection.

The patients included in our study had both a SCVC and a major trauma. The following risk factors were identified in the various studies on trauma patients: older age (>40 or >60 years old), transfusion, surgery, spinal cord injury, pelvic fracture, head injury, and mobility score. Our patients were particularly vulnerable to DVT. First, the patients were in an ICU for severe polytrauma and during the first week they were all immobilized by sedation to allow iterative surgical treatment and mechanical ventilation. Secondly, 70% of these patients had severe brain injury. A brain injury was demonstrated to increase threefold to fourfold the risk of developing a clinically diagnosed DVT, whatever the pharmacological prophylaxis, in two large cohorts of patients admitted to trauma centers [8,20]. In a study on 677 moderately and severely brain-injured patients monitored by duplex scans, a femoral DVT was present in 24% and an upper extremity DVT in 16%. The prevalence of SCVC was not detailed and the authors did not study incidence by severity level of the brain injury [21]. In our patients, brain injury without ICHT was not associated with a higher rate of SCVC-related DVT, whereas ICHT increased the risk fivefold and this association had not yet been demonstrated. This increase in SCVC-related DVT incidence in case of ICHT may be related to coagulation disorders that occur after head injuries [22,23]. Coagulation activation from cerebral blood has been demonstrated very early after a head trauma [24], probably induced by tissue factor release from the injured brain. Sustained coagulopathy is more frequent in patients with tomographic signs of intracranial hypertension (compressed cisterns or midline shift) [25]. In other studies, fibrin degradation products levels were demonstrated to be related to the severity of brain damage [22].

SCVC-related DVT is a particularly underestimated complication. Clinical signs of SCVC-related thrombosis are neither sensitive nor specific [2,5,26,27]. In polytraumatized patients the diagnosis of thrombosis is especially difficult in view of the edema related to their orthopedic injuries. In a study of 208 patients with femoral or subclavian CVC screened by ultrasound in an ICU, 33% of the patients developed CVC-related DVT but none were symptomatic [19]. This was the case in our study. The clinical screening therefore appears ineffective. On the other hand, ultrasound screening is a noninvasive efficient bedside tool, as already demonstrated in orthopedic surgery [28,29]. The clinical importance of asymptomatic DVT remains to be demonstrated. PE occurred in 0 to 17% of cases after an upper-arm DVT in two studies [30,31]. In both studies, however, 10 to 20% of patients had a neoplasm and the responsibility of the catheter is thus difficult to determine. In the RIETE registry of patients with symptomatic upper-extremity DVT or PE, recurrent PE occurred in 3.8% of patients with cancer and 1.6% in those without [32]. In all these cases, however, the DVT was symptomatic.

The second complication may be a post-thrombotic syndrome. Prandoni and colleagues found a 17% cumulative incidence of post-thrombotic syndrome in patients with vein catheter at 1 year but their study concerned only symptomatic upper-arm DVT [33]. Few studies have reported data on the incidence and the range of estimates is wide, reflecting the heterogeneity of patients studied [34]. Whether SCVC-related DVT patients have the same risk of developing long-term post-thrombotic syndrome as other DVT patients is not clear. All of these complications have been studied in symptomatic patients. The complications that may be linked to asymptomatic SCVC-related DVT remain to be confirmed. In Malinoski and colleagues' study, DVT was diagnosed by systematic ultrasound examination and the rate of PE after catheter-associated upper extremity DVT was 1.3% despite generalized thromboprophylaxis [35]. In our study, a diagnosis of PE was confirmed twice. One PE occurred 20 days later than the occurrence of the SCVC-related DVT and was associated with a lower-limb DVT; both DVTs had disappeared when the patient left the ICU. The involvement of the SCVC-related DVT was uncertain. In the second case, the patient was free of DVT at the end of the ICU stay and PE occurred later. The DVT was found near the site of SCVC placement. Three other patients experienced brief episodes of hypoxia when the SCVC was removed. Although these events may seem anecdotal, we think that clinicians should keep in mind the potential harmfulness of SCVC-related DVTs.

High-risk patients should benefit from improved preventive measures. Clinicians are hesitant to use chemoprophylaxis because of the risk of intracranial hemorrhage. LMWH has proved effective as pharmacologic thromboprophylaxis in ICU trauma patients [2,9,17]. However, a recent study demonstrated that pharmacologic thromboprophylaxis use is associated with a 13-fold increased odds of further hemorrhage progression in patients whose follow-up computed tomography within 1 day of admission showed an intracranial hemorrhage increase [36]. Patients at high risk of SCVC-related DVT but presenting a high risk of bleeding could thus not benefit from the anticipated pharmacologic thromboprophylaxis. The benefit of therapeutic anticoagulation was not demonstrated in our patients who underwent examinations after SCVC-related DVT had been diagnosed. At the time of the study, we were very careful about prescribing anticoagulants. When prescribed, the decision was made on the basis of the extent of the thrombosis, and the severity of intracranial, hepatic or pulmonary hemorrhagic contusions. The doses were possibly underestimated in patients with a severe inflammatory condition. However, in the study by Malinoski and colleagues on surgical and trauma patients, anticoagulation was not associated with a higher rate of resolution [35]. Our decision to remove the SCVC was also taken on a case-by-case basis according to the extent of the thrombosis and according to the technical possibility of using another infusion mode. One of our main considerations was not to send a significant clot into the blood flow.

These findings have led us to implement preventive measures to decrease SCVC thrombosis. An educational film about good practices in CVC insertion has been made, which is now available for both junior and senior practitioners. Second, any SCVC whose tip is misplaced in the internal jugular vein or in the innominate vein is removed. The use of ultrasound for SCVC placement decreases the number of punctures and the failure rate, and is now encouraged [37,38]. Third, Duplex ultrasound examinations are now performed by most physicians in our service. They are systematic in all patients at the end of the first week since our study demonstrated that 60% of DVTs occurred within this delay, and then before removing the SCVC. Last, the time of chemoprophylaxis is discussed earlier, on a case-by-case basis, taking into account the risk of intracranial bleeding propagation. At the beginning of the study, thromboprophylaxis was prescribed late. Nowadays, we assess the risk-benefit ratio of beginning prophylaxis much earlier.

## Conclusion

This study demonstrates that SCVC-related DVT is a frequent and early complication in severe polytraumatized patients, and its incidence does not depend on the type of polyurethane. SCVC-related DVT concerns one-third of patients and occurs early within the first week. The main risk factors were intracranial hypertension, a misplaced SCVC, or a massive transfusion. SCVC-related DVT is often underdiagnosed because it is mostly asymptomatic. Further large prospective studies are required to confirm our findings, to identify other risk factors, to study long-term morbidity linked to these SCVC-related DVTs and to evaluate the cost-effectiveness of systematic screening by venous ultrasound in these patients in whom pharmacologic thromboprophylaxis may be hazardous.

## Key messages

• The incidence of SCVC-related DVT does not depend on the kind of polyurethane.

• The main risk factors are intracranial hypertension and misplaced SCVC.

• One-half of CVC-related DVTs were found at the end of the first week after trauma.

• Routine coagulation tests do not help to diagnose these DVTs.

• The long-term consequences of these asymptomatic SCVC-related DVTs would be investigated to determine their clinical impact.

## Abbreviations

AIS: Abbreviated Injury Score; Al: aliphatic polyurethane; Ar: aromatic polyurethane;CVC: central venous catheter; DVT: deep venous thrombosis; ICHT: intracranial hypertension;IQR: interquartile range; ISS: Injury Severity Score; LMWH: low molecular weight heparin; PE: pulmonary embolism; SCVC: subclavian central venous catheter.

## Competing interests

The authors declare that they have no competing interests.

## Authors' contributions

LP, FM, VC, JB-B and GF contributed to the study conception. AG, LP, FM, VC and FS contributed to the data acquisition, and writing. LP and VC performed ultrasonographic examinations. CP, AL and J-FC contributed to clinical examinations of patients to determine the probability of thrombosis. FM contributed to the data analysis. All authors have made substantial contributions to the conception, the design and interpretation of results, have been involved in drafting the manuscript or revising it critically for important intellectual content, and have given final approval of the version to be published.
